# Frequency of Fimbrial Gene Types I, Ib, and II in Clinical Strains of Porphyromonas gingivalis Characterized From Periodontitis Patients

**DOI:** 10.7759/cureus.64117

**Published:** 2024-07-08

**Authors:** Pradeep V R., A. S. Smiline Girija, J. Vijayashree Priyadharsini, K. Kannika Parameshwari

**Affiliations:** 1 Department of Microbiology, Saveetha Dental College and Hospitals, Saveetha Institute of Medical and Technical Sciences, Saveetha University, Chennai, IND

**Keywords:** stage iii periodontitis, oral health, periodontitis, fimbriae genes, porphyromonas gingivalis

## Abstract

Objective

*Porphyromonas gingivalis* (*P. gingivalis*) is considered the predominant pathogen in association with different stages of periodontitis, and fim genes play a vital role in adherence and colonization. This study is thus aimed to detect the prevalence of *P. gingivalis* and the frequency of fim gene types among the clinical strains isolated from periodontitis patients.

Methods

Plaque samples (N = 45) were collected from patients with three different stages of periodontitis (n = 15 in each group). All the samples were inoculated onto sterile anaerobic blood agar and were processed anaerobically using a GasPak system at 37°C for five to seven days. Standard microbiological techniques were used to identify *P. gingivalis*. Genomic DNA was extracted, and polymerase chain reaction (PCR) was carried out to detect the frequency of three fim gene types, using specific primers.

Results

*P. gingivalis* was more prevalent in Group III (93.3%), followed by 26.7% in Group II, and 13.3% in Group I. Maximum isolates were seen in the age group of 40-50, with no significance within the genders. fim type I was frequent in Group III (78.5% (n = 11)), followed by 0.25% (n = 1) under Group II, with no other fim types in the other groups.

Conclusion

Prevalence of *P. gingivalis* and frequency of fim genes, in association with its virulence, were observed. Periodical monitoring of such virulence genes would aid in the theranostic approach to combat the complications caused by *P. gingivalis* in periodontitis cases.

## Introduction

Periodontitis is an inflammatory condition of the structures around the tooth, caused by the polymicrobial conglomeration of microbes. Periodontitis occurs in different stages and is clinically graded based on its pathology [1]. The first stage, gingivitis, is the most prevalent and earliest type of periodontal disease, manifesting as inflammation of the gingiva. It further progresses to the second stage, which is referred to as early or mild periodontitis, initiating bone loss around the tooth. The third stage is moderate periodontitis; as the condition worsens from mild to moderate, infection and inflammation penetrate deeper into the tissues, causing 20-50% of the bone surrounding the tooth root to disappear. The final stage is aggressive or advanced periodontitis, resulting in extreme bone loss, defined as the loss of 50-85% of the tooth root's bone support, causing teeth to become extremely loosened and unstable during functional movements [2]. Periodontitis is also linked to a number of systemic disorders, including problems in the reproductive, respiratory, musculoskeletal, and cardiovascular systems, and oral microbial dysbiosis in periodontitis, with the obligate anaerobes attributing to the progression of the disease [3]. The predominant pathogens encountered are *Porphyromonas gingivalis* (*P. gingivalis*), *Treponema denticola*, and *Tannerella forsythia.*

Among these, *P. gingivalis* and aggressive periodontitis have consistently been linked in many of the earlier studies [4]. *P. gingivalis* plays a significant role by upsetting host immunological homeostasis, contributing to the etiology of periodontitis [5]. It is well known that *P. gingivalis*, a black-pigmented, gram-negative anaerobic rod, is abundant in the sub-gingival biofilms, playing a phenomenal role in the establishment of periodontal diseases, together with other oral pathogens. Although the gingival sulcus and periodontal pocket are the main ecological niches of *P. gingivalis*, the organism may come into contact with any of the oral surfaces influenced by host-associated factors [6]. *P. gingivalis* has the ability to cling onto various surfaces and frequently exhibits multimodal adhesion mechanisms designed to boost the avidity of binding and the likelihood of attachment [7]. It is also noticed that grade III periodontitis may be brought on by specific virulent strains of the pathogen, producing vital virulence factors. Amidst various virulent determinants, fimbriae, cysteine proteinases, hemagglutinins, and lipopolysaccharide (LPS) are known to be associated with its pathogenesis [8].

It is a known fact that the proteinaceous appendages are frequently seen on the surfaces of bacteria, for it to attach and invade the host cells, and the special type of extracellular polymers or non-flagellar appendages are termed pili or fimbriae [9]. Fimbriae are considered as important because they initiate bacterial adherence and penetration in specific locations, which is vital for colonization in the sub-gingival regions by *P. gingivalis.* *P. gingivalis *expresses two different and distinct fimbrial structures on its cell surface. The fimbrial protein is made up of a subunit protein called fimbrillin which is encoded by the fimA genetic determinant. One type of fimbriae is long and comprises a subunit Mfa protein encoded by the mfa1 gene. The other fimbria is short or minor and is a homopolymer component of the protein with a molecular mass of 75 kDa. The antigenicity of both the fimbriae varies in both its structure and function. The *P. gingivalis *chromosome only contains one copy of the fimA gene, which encodes for fimA. Studies on the fim genes categorize them into six types (I, Ib, II, III, IV, and V) based on variations of their nucleotide sequence. FimA is a unique category of fimbriae distinct from the types I and IV families, and it possesses an unusually lengthy signal peptide requiring Arg- and Lys-specific proteases (gingipains) for extracellular maturation [10].

It is thus highly essential to assess the expression of fimbriae genes to understand the virulence and potentiality of *P. gingivalis* to progress to severe grades of periodontitis. This study is thus aimed to molecularly characterize the different types of fim gene types from the clinical isolates of *P. gingivalis*, characterized by patients with aggressive periodontitis.

## Materials and methods

Sampling and microbiological processing

Periodontal patients (N = 45) visiting Saveetha Dental College and Hospitals, Chennai, India, were categorized into three groups: Group 1: gingivitis, Group 2: stage II periodontitis, and Group 3: stage III periodontitis. Informed consent and ethical clearance were obtained prior to initiating the study (Ref: SRB/SDC/UG-2113/23/MICRO/128). The subjects had not taken any antibacterial medication or undergone professional cleaning in the three months before the study. Plaque samples were collected using a sterile curator and were placed in sterile tryptic digest blood broth supplemented with sheep blood (5%), vitamin K (5%), and hemin (5%). The samples were immediately transferred to the microbiology lab for processing using an anaerobic cultivation method.

Isolation of *P. gingivalis*

Plaque samples were plated onto sterile anaerobic blood agar with sheep blood (5%), hemin (5%), and menadione (5%). The samples were placed in an anaerobic jar with a gas pack system after being streaked onto recently prepared sterile blood agar plates. For five to seven days, the plates were incubated at 37°C. After incubation, the plates were observed for the presence of black-pigmented colonies. Preliminary identification of the morphology was conducted using gram staining. Genomic DNA was isolated using the DNA isolation kit as per the manufacturer’s instructions (Puregene; Gentra Systems, Minneapolis, MN, USA) and was stored at 4°C until further processing.

Molecular characterization of *P. gingivalis*

Polymerase chain reaction (PCR) was carried out to confirm the strains of *P. gingivalis *and fimtypes I, Ib, and IIusing specific primers (Table 1). The PCR conditions were set as follows: a five-minute denaturation at 95°C, 30 cycles of 94°C, 58°C, and 72°C, and a seven-minute final extension at 72°C. The PCR products were electrophoresed using a 1% agarose gel, Tris-acetate, and ethylenediaminetetraacetic acid (EDTA) buffer. The amplicon size was observed and the size was compared and confirmed with a 100-bp DNA ladder (New England Biolabs, Beverly, MA, USA). 

**Table 1:** Primer details and amplicon size of fimA types in P. gingivalis selected for the study *P. gingivalis*:* Porphyromonas gingivalis*

Fim types	Primers	Sequence	Product (bp)
P. gingivalis	-	F: ACCTTACCCGGGATTGAAATG R: CAACCATGCAGCACCTACATAGAA	88
Type I fimA	FimA I	F: 5′CTG TGT GTT TAT GGC AAA CTT C 3′ R: 5′AAC CCC GCT CCC TGT ATT CCG A 3′	392
Type Ib fimA	FimA Ib	F: 5′CAG CAG AGC CAA AAA CAA TCG 3′ R: 5′TGT CAG ATA ATT AGC GTC TGC 3′	271
Type II fimA	FimA II	F: 5′ACA ACT ATA CTT ATG ACA ATG G 3′ R: 5′AAC CCC GCT CCC TGT ATT CCG A 3′	257

## Results

**Figure 1:**
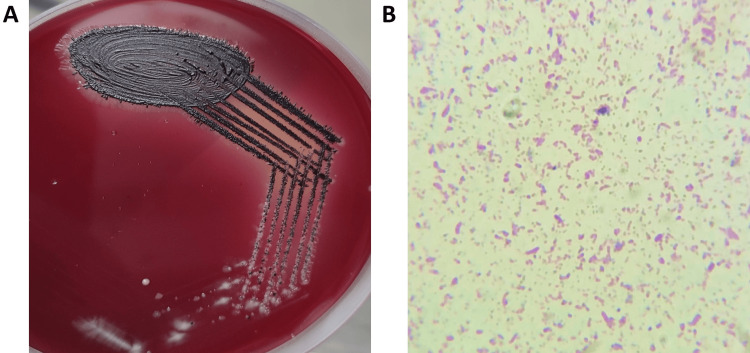
(A) Typical black pigmented colony morphology of P. gingivalis in anaerobic blood agar plate and (B) gram staining showing the pleomorphic gram-negative bacilli *P. gingivalis*: *Porphyromonas gingivalis*

Out of 45 samples (N) across the three groups, 20 samples (62.5%; N = 45) showed the growth of *P. gingivalis*, with 14 isolates (93.3%) from Group III (n = 15), followed by four isolates (26.7%) from Group II (n = 15), and two isolates (13.3%) from Group I (n = 15). The colonies were typically observed as colonies with black pigments, and the gram-negative bacilli showed pleomorphism with gram staining (Figure 1).

**Figure 2:**
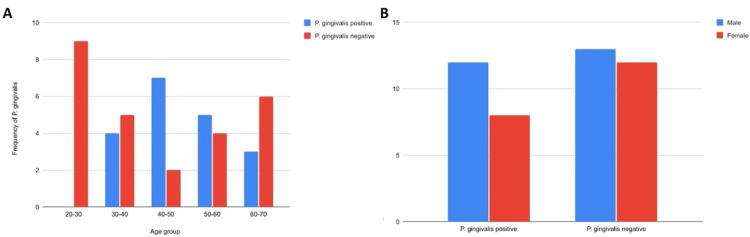
Prevalence of P. gingivalis in different age groups and gender selected for the study *P. gingivalis*: *Porphyromonas gingivalis*

Plaque samples from patients in the age group of 20-30 showed no growth of *P. gingivalis*; however, maximum isolates (n = 7) were observed in the age group of 40-50, followed by five isolates in the age group of 50-60 and four isolates from the age group 30-40, and three in the age group of 60-70 (Figure 2A). Out of the 45 study cases, 25 were male patients and 20 were female patients, in which 48% (n = 12) and 40% (n = 8) showed the presence of *P. gingivalis* in males and females, respectively (Table 2), with no significant difference in the prevalence between the genders (Figure 2B).

**Table 2:** Prevalence of P. gingivalis and frequency of fimA gene types among the study groups *P. gingivalis*: *Porphyromonas gingivalis*

Groups	Sample size (n)	Prevalence of *P. gingivalis*	FimA type I	FimA type Ib	FimA type II
Group 1 - Gingivitis	15	2 (13.33%)	-	-	-
Group 2 - Periodontitis	15	4 (26.66%)	1 (25%)	-	-
Group 3 - Aggressive periodontitis	15	14 (93.33%)	11 (78.5%)	-	-

**Figure 3:**
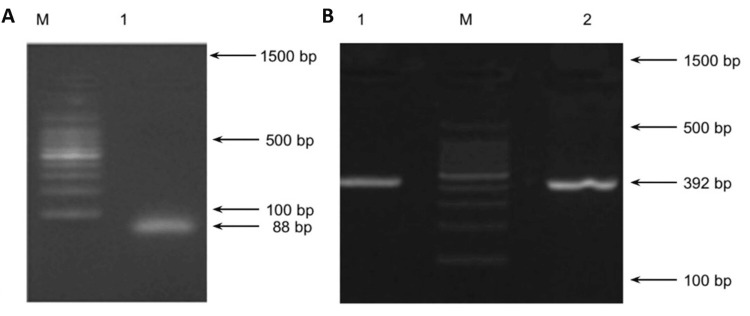
Agarose gel electrophoretogram showing (A) genotypic confirmation of P. gingivalis (amplicon size 88 bp); (B) amplification of fimA type I (amplicon size 392 bp) run along with standard DNA ladder (lane M = 100 bp DNA marker) *P. gingivalis*: *Porphyromonas gingivalis*

The frequency of fim gene type 1 was observed with 78.5% (n = 11) from the 14 isolates in Group III, followed by one isolate (0.25%) in Group II and no fim types in Group I. The amplicon size was observed to be 392 bp (Figure 3). The other types of fim genes were not detected in any of the isolates from all the study groups (Table 2).

## Discussion

Periodontitis, often referred to as gum disease, is an inflammatory condition of the soft tissues and supporting structures of the tooth. Various periodontal pathogens have been reported to be associated with periodontitis, but numerous researchers have identified *P. gingivalis* as one of the main causal agents. The oral cavity encompasses various functional biomes [11]. *P. gingivalis* is frequently linked to chronic periodontitis through bacterial dysbiosis [12]. The virulence factors of *P. gingivalis* play crucial roles in its colonization and the pathogenesis of periodontitis [13]. Studies on host-*P. gingivalis* interactions show that adhesive properties, biofilm formation, and loss of alveolar bone are mainly aggravated by the rapid colonization that occurs through its fimbriae [14]. The long and short fimbriae of *P. gingivalis* are extensively studied [15,16]. In this context, we have chosen *P. gingivalis* to be assessed in association with different stages of periodontitis and the prevalence of fim gene types from clinical isolates.

The findings of our study clearly show a higher prevalence (93.33%) of *P. gingivalis* in stage III (aggressive periodontitis) cases. Our findings also suggest that the frequency of fimA genes is higher in patients with aggressive periodontitis. None of the other fimbriae genes were noticed in the samples analyzed by our study. The fimA gene was observed by PCR with an amplicon size of 392 bp, thus it was categorized as fimA type I. In contrast, earlier studies have shown the frequency of fimA type II from *P. gingivalis* in association with gingivitis conditions. In another study, the frequencies of types Ib and IV were observed in Germany and Japan [17,18]. Such differences may be due to geographical habitat and host-associated factors.

In correlation with our study, fimA allelic composition and variations have been demonstrated among the clinical strains [19]. The fimA genotype may also be correlated with other virulence-related factors, such as higher elastolytic activity in type I strains [20], increased hemagglutination as evidenced in type IV strains, and increased Nα-benzoyl-dl-arginine 4-nitroanilide hydrochloride (BAPNA) activity in type II strains [21,22].

In the context of age, *P. gingivalis* was more prevalent in the elderly population compared to the younger subjects under study. This correlates with an earlier study where similar age-related observations have been documented [23], with higher prevalence at the mean age group of 51.4 years [24]. Also, there were no significant differences observed in the prevalence of *P. gingivalis* between the genders in our study. A similar study also confirmed that there were no notable variations in *P. gingivalis* prevalence between the sexes [25]. In another study, no such differences in prevalence were noticed between the genders of elderly patients as well [26].

Limitations of the study include a smaller sample size, thus statistical analysis was not performed to report the significance of the isolates and fim genes observed. The study focused only on fim genes; no other virulence factors were studied to detail its virulence. Additionally, using computational approaches, putative epitope peptide predictions being possible [27,28], fim-based genes may be considered as a novel target in designing periodontal vaccines [29]. This will also aid in the development of innovative periodontal therapeutic approaches aimed at specifically targeting such virulent genes in the near future.

## Conclusions

The prevalence of *P. gingivalis* was found to be highest in aggressive periodontitis elderly patients, with no differences observed among the genders. Fim type I seems to be more prevalent among the clinical strains, which could be attributed to the pathogenesis of periodontitis. This is a first-of-its-kind study that documents the frequency of fim types from the clinical strains of *P. gingivalis*. The study further concludes with the need to design novel drugs targeting the fim genes to treat patients with aggressive periodontitis caused by *P. gingivalis*.
